# Exploring the feasibility of a web-based positive psychology program among anaesthesiologists: an explanatory sequential mixed-methods pilot study

**DOI:** 10.1186/s12909-026-09459-2

**Published:** 2026-05-18

**Authors:** Albert Kam Ming Chan, Carl Ming Sung Chung, Ka Chun Wu, Yeung Yan Stacie Hoi

**Affiliations:** 1 Department of Anaesthesia, Pain and Perioperative Medicine, Prince of Wales Hospital, Hong Kong, China; 2 Teengineer – Institute of Positive Psychology, Hong Kong, China; 3 Faculty of Social Sciences, University of Hong Kong, Hong Kong, China

**Keywords:** Positive psychology, Anaesthesiology, Well-being, Physician burnout, Mixed-methods research, Feasibility, Web-based intervention, Psychological safety, Self-determination theory, Hong Kong

## Abstract

**Background:**

Physician burnout is a global crisis, with Hong Kong doctors reporting a personal burnout rate as high as 72%. Anaesthesiology is considered a high-risk specialty, with high risk of depression and suicide. Time constraints preclude engagement of healthcare professionals with structured psychological interventions despite their known benefits. Web-based positive psychology interventions (PPI) offer an accessible alternative for busy clinicians. This mixed-methods study aimed to evaluate the feasibility and acceptability of culturally adapted, web-based PPIs for anaesthesiologists in Hong Kong, and through exploratory efficacy assessment, to explore whether PPIs that are focused on personal growth vs. workplace application may have an effect on well-being.

**Methods:**

An explanatory sequential mixed-methods feasibility randomized controlled trial with exploratory efficacy outcomes was employed for this study. Fellows and members of the Hong Kong College of Anaesthesiologists were randomized (1:1:1) to two experimental groups (Exp1 individual-oriented PPI/Exp2 workplace-oriented PPI) or wait-list control. The intervention consisted of an induction workshop followed by 4 web-based modules with bite-sized exercises and WhatsApp peer discussions over 10 weeks. Primary outcomes (PERMA-Profiler, GAD-7, PHQ-9, PSS-10, Copenhagen Burnout Inventory) and secondary outcomes (SCS-SF, WAMI, WGS) were measured at baseline, post-intervention and one-year follow-up. Semi-structured interviews were conducted to explore feasibility and participant perceptions.

**Results:**

One hundred two participants were recruited for the study. Quantitative outcomes were interpreted as exploratory given the limited sample size. Linear mixed model analysis did not demonstrate significant, sustained improvements in primary outcomes across time points between groups. However, Exp1 showed a significant reduction in depression and increase in meaningful work immediately post-intervention, although this was not sustained at one-year follow-up. Qualitative analysis identified six themes, affirming that the blended web-based approach was feasible. Participants’ intrinsic motivation and the perceived applicability to professional and personal domains were key enablers of PPIs. PPIs facilitated participants’ competence in the face of clinical challenges, and enabled relational building in the workplace. Implementation challenges included waning engagement due to busy schedules and concerns regarding psychological safety within WhatsApp group chats.

**Conclusions:**

Bite-sized, web-based positive psychology interventions appear feasible and acceptable for time-constrained clinicians such as anaesthesiologists in Hong Kong. In this exploratory feasibility trial, PPIs designed to support the basic psychological needs described in self‑determination theory showed preliminary short‑term benefits for well‑being, but future large-scale efficacy trials should evaluate whether such effects can be sustained through complementary system-level measures, including leadership and organizational initiatives that provide autonomy-supportive environments for healthcare professionals.

**Trial registration:**

The study was registered on ClinicalTrials.gov (ClinicalTrials.gov ID: NCT06148454) on November 19, 2023.

**Supplementary Information:**

The online version contains supplementary material available at 10.1186/s12909-026-09459-2.

## Background

Doctors’ well-being is an integral part of quality patient care. Physician burnout has become increasingly recognized as a threat not only to the well-being of medical professionals, but also to healthcare systems and their sustainability globally – this has only been amplified in the face of the COVID-19 pandemic. The World Health Organization classifies burnout as an occupational syndrome characterized by emotional exhaustion, depersonalization, and decreased sense of accomplishment [1]. The magnitude of the problem has been studied worldwide, including the United States of America, the United Kingdom and Australia, citing burnout rates among surveyed physicians of up to 50% [2–7]. It is particularly concerning in Hong Kong, where a recent survey indicated that 72.6% of Hong Kong doctors reported personal burnout [8]. The impact of burnout extends beyond the individual, and in a recent systematic review, has been shown to decrease quality of care, increase medical errors, decrease patient satisfaction, decrease productivity and professional effort, and increase staff attrition [9]. The financial implications are also very concerning, with physician burnout costing the U.S. healthcare system an estimated US$4.6 billion annually [10]. This is an imminent threat that must be addressed promptly.

Anaesthesiology has been regarded as one of the more burnout-prone medical specialties in the literature, with a high risk of depression and physician suicide [11]. In the USA, the reported prevalence of attending anaesthesiologists who are at high risk of burnout ranges from 39.7 to 59.2%, compared with 43.9% of all physicians [12]. It has been suggested that anaesthesiologists are at high risk as they constantly need to make quick and astute clinical decisions when managing critically ill patients and crises [13, 14]. This is exacerbated by chronic and extreme stress associated with frequent night shifts and specialty examinations [13, 14]. In a cohort of anaesthesiologists studied, over 80% experienced perioperative catastrophes that significantly affected their psychological well-being [15]. Moreover, anaesthesiology also carries substantial malpractice risks, ranking among the top five for litigation claims in healthcare systems [16]. It is imperative to identify effective measures to counter burnout among anaesthesiologists.

Structured psychological interventions have demonstrated promise in reducing physician burnout and enhancing resilience [17–20]. One example is positive psychology – the scientific study of character strengths, positive relationships and life purpose that enable individuals and communities to thrive and live meaningful lives. The theoretical components of positive psychology are incorporated in PERMA (positive emotions, engagement, relationships, meaning, accomplishment) by Martin Seligman [21]. Through interventions such as gratitude, positive goal setting, optimistic thinking, acts of kindness, mindfulness and meaningful activities, positive psychology has been associated with improvement in subjective and psychological well-being [22–25]. A recent systematic review on positive psychology interventions in workplace settings showed a moderate effect on well-being (e.g. life satisfaction, personal growth) and self-reported performance (perceived contribution, energy at work, organizational virtues), and these effects may persist over time [26]. Moreover, mindfulness‑based and other positive psychology interventions show preliminary evidence of reducing stress, depressive and anxiety symptoms, and burnout among healthcare workers, with modest improvements in well‑being and some empathy‑related outcomes [27–30]. Another recent systematic review showed that system-directed and workplace-oriented PPIs that value enabling individuals to commit and find meaning in the profession, may produce more favourable results compared to individual physician-directed PPIs [31] – which calls for the design of more specific PPIs for practicing clinicians.

However, conventional delivery models of psychological interventions require extensive time commitment, such as in the form of face-to-face meetings or workshops, which physicians often find difficult in the face of busy clinical work and commitments. Time constraints are frequently listed as one of the major barriers for physicians seeking psychological support [32, 33]. The ubiquitous nature of web-based applications can potentially provide highly accessible, convenient and anonymous way to reach healthcare professionals who would otherwise not have time to seek help due to inconvenience, stigma and other help-seeking barriers. This has been demonstrated in the field of clinical psychology, where mobile web-based mindfulness training, self-compassion training and cognitive behavioural psychoeducation have been efficacious in improving mental well-being and reducing psychological stress [34–36]. Recent literature has also demonstrated the role of web-based interventions in improving the well-being of healthcare professionals [37–40].

Despite evidence in the literature showing promise regarding the effectiveness of PPIs in reducing burnout and promoting well-being among healthcare professionals, their application and efficacy among anaesthesiologists in Hong Kong are largely unknown. While Hong Kong doctors experience high rates of burnout, local research on targeted intervention strategies, such as PPIs, is lacking. The cultural and contextual factors unique to Hong Kong’s healthcare system, including high patient loads, resource constraints, and cultural attitudes toward mental health, may influence the acceptability and effectiveness of these interventions.

To address these challenges, our research team developed an innovative web-based positive psychology programme tailored for Hong Kong anaesthesiologists. The intervention merges a concise, half-day induction workshop followed by flexible web-based components and peer discussions, with the aim of overcoming scheduling barriers while preserving interactive elements to enhance engagement.

The aim of the mixed-methods feasibility randomized controlled trial was to (1) evaluate the feasibility and acceptability of web-based PPIs among anaesthesiologists (2) explore contextual and motivational factors influencing participation (3) conduct exploratory assessment of efficacy to generate preliminary effect estimates to inform the design of future trials, particularly in time‑constrained medical specialties.

## Methods

### Setting

This study took place between November 2023 and November 2025 with the Hong Kong College of Anaesthesiologists (HKCA). HKCA is the statutory body under the Hong Kong Academy of Medicine responsible for training, examination and accreditation of anaesthesiologists in the territory. Its membership encompasses the entirety of the anaesthesia workforce in Hong Kong, including all trainees and fellows in both public and private practice.

### Participants

Participants were recruited through email circulated by HKCA. To be eligible for the study, the participants must be a fellow or member of HKCA. All participants provided informed consent, and anonymity was ensured in data handling and reporting.

### Study design

An explanatory sequential mixed-methods study design was adopted [41]. Participants were assigned in a 1:1:1 ratio to Experimental Group 1 (Exp1), Experimental Group 2 (Exp2), or the waitlist control group (Control). The three-arm design was chosen to compare individual growth-oriented PPI (Exp1), workplace-oriented PPI (Exp2) and control. Recruitment and interventions were conducted in two cohorts: Batch 1 from November 2023 to January 2024, and Batch 2 from August 2024 to October 2024. Planned a priori quantitative data collection and analysis using validated outcome measures were determined before the commencement of the study, and these data were then used to inform the development of a semi-structured interview guide for subsequent qualitative data collection and analysis.

The explanatory sequential mixed-methods design was chosen specifically because, although standardized self-report instruments are essential for generating comparable, psychometrically robust outcome data, they often fail to capture the nuanced, contextualized reasons why complex educational and psychological interventions do or do not work for the participants. The trial was therefore designed as a feasibility randomised controlled trial – with the quantitative limb assessing feasibility and acceptability and providing exploratory estimates of intervention effects, and the qualitative limb elaborating mechanisms, contextual influences, and participant experiences to provide a more comprehensive and practically meaningful evaluation of web‑based PPIs for anaesthesiologists in Hong Kong [42].

The study was registered on ClinicalTrials.gov (ClinicalTrials.gov ID: NCT06148454) on November 19, 2023. Ethical approval was granted by the Chinese University of Hong Kong Survey and Behavioural Research Ethics Committee on October 17, 2022. (Ref.: SBRE-22-0147).

### Intervention

Four psychoeducation modules were developed to teach the physicians about the concepts of positive psychology and coping skills to challenges in their work and personal lives based on established positive psychology theories [21, 43–46]. Each experimental group received two thematic modules, with the intervention spanning approximately four to five weeks per module. Within each module, participants completed five to seven web-based exercises, each designed to take no longer than 15 min to complete. The exercises followed a tripartite structure, combining instructional content, exercises, and reflection prompts:

- Bite-sized theories: Selected theoretical content was presented in multiple engaging formats—including short videos, infographics, brief written articles, and interactive games. The multidimensional presentation was intended to maximize accessibility and accommodate diverse learning preferences. The web-based content was developed and delivered via the Qualtrics platform software (Qualtrics, Provo, UT).
- Application: Following the theoretical overview, participants undertook practical exercises requiring the application of learned concepts in daily clinical or personal contexts. This emphasis on experiential practice reflected the intention to bridge theory and the real-world challenges faced by anaesthesiologists.
- Sharing: After completing the exercises, participants were prompted to reengage with their WhatsApp group, where they shared reflections and emotional responses. This peer-to-peer sharing was designed to foster connection, normalize experiences, and promote mutual encouragement, consistent with evidence that social support is a key determinant of psychological well-being and resilience among healthcare professionals [47].

As individual-oriented PPIs and workplace-oriented PPIs were of equal theoretical and practical interest in light of prior evidence [31], four modules were conceptually organized into these two corresponding domains Specifically, Experimental Group 1 (Exp1) received *Self-awareness & Positivity* plus *Compassion & Leadership* that focused on individual and personal growth, while Experimental Group 2 (Exp2) received *Rapport & Conflict Management* plus *Meaning & Accomplishment* that focused on workplace-related application. Table 1 provides a summary of the weekly content of each module.

**Table 1:** Weekly summary of the psychoeducation modules

Module	Self-awareness & Positivity	Compassion & Leadership	Rapport & Conflict Management	Meaning & Accomplishment
Week 1	Understanding Psychological Well-being	Foundations of Self-Compassion	Understanding Workplace Relationships	Savoring
Week 2	Emotional literacy	Myths about Compassion	Managing emotions at conflict	Professional Identity and Achievement
Week 3	Positive self-talks	Self-Acceptance	Emotional maturity	Meaning and purpose
Week 4	Gratitude practice	Sense of Humanity	Active listening	Work-Life Harmony
Week 5	Gratitude	Compassionate Leadership	High Quality Connections	Cultivating sense of victory
Week 6	Quality rest	Compassionate actions at Workplace	Adapting to Different Mindsets	Good goals and Goal-setting
Week 7	Integrating positivity and emotional awareness	Integrating Compassion into Leadership Practice	Integrating Emotional and Interpersonal Skills	Integrating Meaning and Sustained Growth

Cultural adaptations of the PPIs were made to cater for the nature of anaesthesia practice in Hong Kong. Specifically, the exercises were formatted as bite-sized practices to accommodate clinician’s heavy workload; and the content of PPIs, such as positive self-talk, cognitive reappraisal and self-compassion, was explicitly tailored to address the characteristics of anaesthesiologists including perfectionism, self-denial and low tolerance of errors [48]. These adaptations were directed primarily at the professional culture of anaesthesiology (work patterns, role expectations and norms around error and self‑criticism) within the broader healthcare context of Hong Kong, rather than the national culture per se.

To support intervention fidelity, each intervention group had a dedicated WhatsApp group in which the facilitator posted standardised, scheduled reminders when new exercises were released and prompted participants to share brief reflections approximately every two weeks. Participant adherence was monitored via engagement in these WhatsApp discussions (number and frequency of posts) and completion data from the Qualtrics platform for each exercise and module. Standardisation across participants was ensured by delivering all content through pre‑designed Qualtrics modules; all participants within the same experimental arm received the same sequence of materials, instructions, and reflection prompts.

### Quantitative component

#### Participants and procedure

Participants were randomized into one of the three arms: Exp1, Exp2, and Control. The two experimental groups received two different psychoeducation curricula, while the Control received no active intervention during the intervention period.

**Fig. 1:**
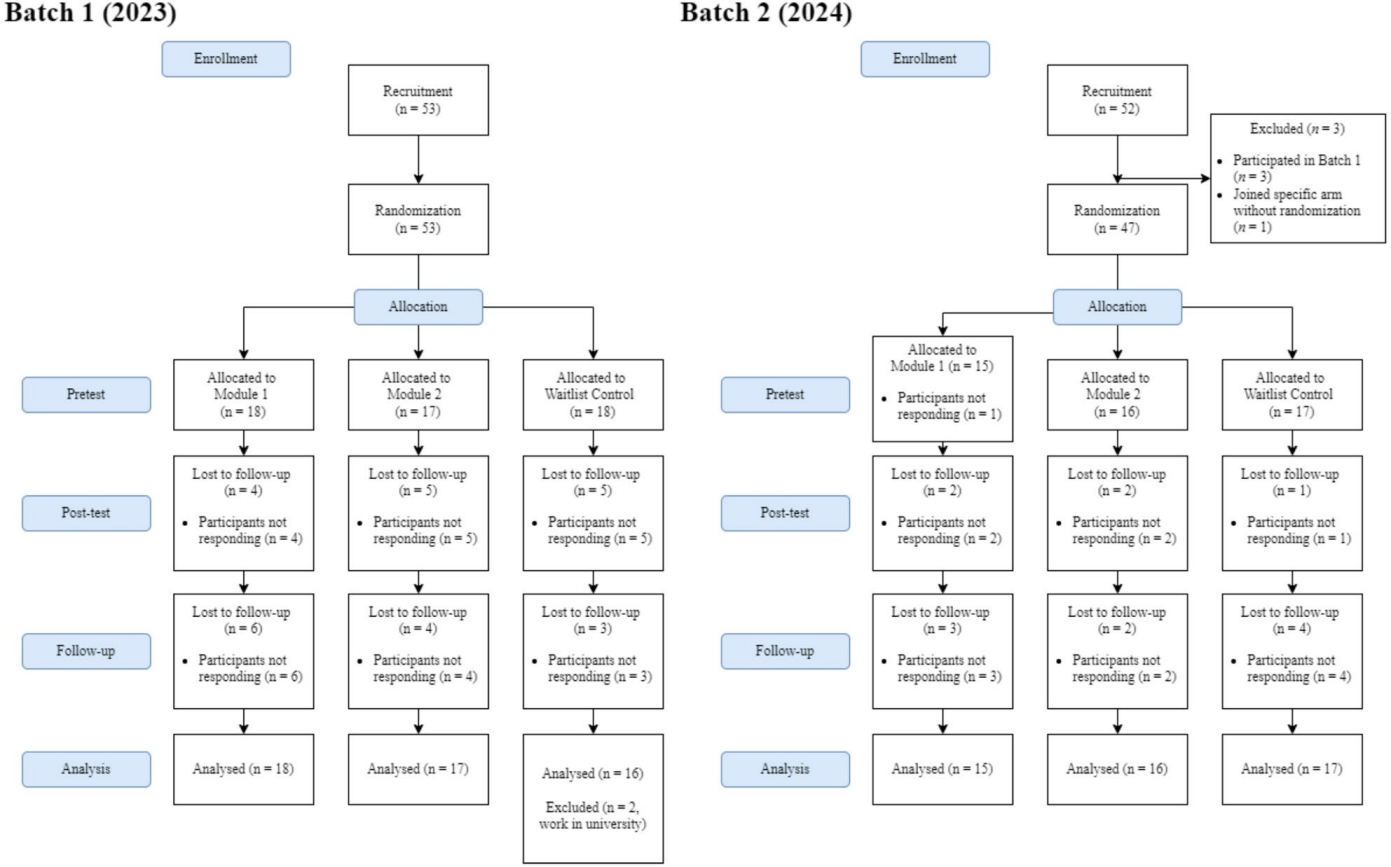
CONSORT flow diagram

The blockrand package was used to create permuted blocked randomisation sequences [49, 50], stratified by practice setting (Public, Private, University) and professional status (Fellow, Trainee). The participant flow of the two cohorts is presented in Fig. 1, and their demographics are presented in Table 2.

**Table 2:** Demographics of study participants

	Batch 1 (*n* = 53)			Batch 2 (*n* = 48)			Total (*N* = 101)		
	Exp1 (*n* = 18)	Exp2 (*n* = 17)	Control (*n* = 18)	Exp1 (*n* = 15)	Exp2 (*n* = 16)	Control (*n* = 17)	Exp1 (*n* = 35)	Exp2 (*n* = 33)	Control (*n* = 33)
Gender									
Male	12	11	9	4	6	4	17	16	22
Female	6	6	9	11	10	13	16	17	13
Membership ^a^									
Fellow	14	13	15	8	8	11	22	21	26
Trainee	4	4	3	7	8	6	11	12	9
Practice ^a^									
Public	13	12	11	11	11	12	24	23	23
Private	5	5	6	4	4	5	9	9	11
University	0	0	1	0	0	0	0	1	1

Exp1 = Experimental group 1; Exp2 = Experimental group 2

^a^ Variables used for balancing in the stratified randomization procedure

#### Sample size

An a priori power calculation for a three-arm design indicated that a total sample of approximately 324 participants (108 per group) would be required to detect a medium effect size in the primary outcome with 80% power at alpha = 0.05 [23]. Given the finite pool of eligible anaesthesiologists (~ 500 active fellows and ~ 200 trainees) and the competing demands of full-time clinical duties, examination preparation and service pressures, achieving this target was unrealistic. As such, the trial was designed pragmatically as a feasibility study and was not powered to detect small or medium between‑group effects. Inferential analyses of psychological outcomes were conducted as exploratory efficacy assessments, intended to yield preliminary estimates of potential intervention effects to inform the design and sample size of future, adequately powered trials. Accordingly, we adopted a pragmatic feasibility approach and aimed to recruit as many participants as possible within the study time frame.

#### Outcome measures

Quantitative outcomes were measured via online questionnaires administered at three time points: baseline (T1), post-intervention (T2), and one-year follow-up (T3). The baseline measures were collected one week before the intervention period, prior to randomization. Post-intervention measures were collected within two weeks after the intervention period ended. Follow-up measures were collected one year after baseline. Web-based modules completion data for each experimental group was captured via the Qualtrics platform.

#### Primary outcomes

##### Well-being

The 23-item PERMA-Profiler [51]was used to measure the five pillars of well-being defined by Seligman [21]: Positive emotion, Engagement, Relationships, Meaning, and Accomplishment. Each domain contained three items with eight additional items assessing overall happiness, negative emotion, loneliness, and physical health. Participants responded on an 11-point Likert scale ranging from 0 = “Never/not at all” to 10 = “Always/completely”. An overall well-being score was calculated by averaging all items from the five subscales and the overall happiness item.

##### Perceived stress

The Perceived Stress Scale (PSS-10) was used to measure participants’ stress [52]. Participants were asked how often they felt stressed over the last month. Participants responded to ten items on a 5-point Likert scale, ranging from 0 = “Not at all” to 4 = “Very often”. After reversing the scores of items 4, 5, 6, and 8, the total score of all items was calculated, with a higher score indicating more severe stress.

##### Anxiety

The Generalized Anxiety Disorder 7-item (GAD-7) was used to measure participants’ anxiety symptoms [53]. The scale measured the frequency of the participants experienced anxiety symptoms over the last two weeks and responded to seven items on a 4-point scale, ranging from 0 = “Not at all” to 3 = “Nearly every day”. A total score of all items was calculated, with a higher score indicating more severe anxiety.

##### Depression

The Patient Health Questionnaire (PHQ-9) was used to measure participants’ depressive symptoms [54]. The scale measured the frequency of the participants experienced depressive symptoms over the last two weeks and responded on a 4-point scale, ranging from 0 = “Not at all” to 3 = “Nearly every day”. A total score of all items was calculated, with a higher score indicating more severe depression.

##### Burnout

The Copenhagen Burnout Inventory (CBI) was used to measure participants’ state of prolonged physical and psychological exhaustion across three domains: personal burnout, work-related burnout, and client-related burnout [55]. Participants responded on a 5-point Likert scale, mapped to 0-100 (ranging from 0 = “Never/almost never” to 100 = “Always”). After reversing the score of the last item of the work-related burnout subscale, the total score of each subscale was calculated, with a higher score indicating more severe burnout.

#### Secondary outcomes

##### Self-compassion

Self-Compassion Scale Short Form (SCS-SF) was used to measure participants’ ability to hold one’s feelings of suffering with a sense of warmth, connection and concern [56]. Participants responded on a 5-point Likert scale, ranging from 1 = “Almost never” to 5 = “Almost always”. A total self-compassion score was calculated by reversing the scores of the negative items and averaging all items.

##### Meaningful work

The Work and Meaning Inventory (WAMI) was used to measure the extent to which a person perceives work as a meaningful experience [57]. Participants responded on a 5-point Likert scale, ranging from 1 = “Absolutely untrue” to 5 = “Absolutely true”. An overall meaningful work score was calculated by reversing the scores of the negative items and summing the scores of all items.

##### Work-related gratitude

The Work Gratitude Scale (WGS) was used to assess the deliberate attitude of engaging in positive appraisal and feeling of thankfulness at work [58]. Participants responded on a 7-point Likert scale, ranging from 1 = “Strongly Disagree” to 7 = “Strongly Agree”. An overall gratitude at work score was calculated by taking the mean score of all three components (grateful appraisals, gratitude toward others, intentional attitude of gratitude) in a 10-item multidimensional measure.

#### Post-intervention feedback

At post-test, additional open-ended questions were asked to capture the participants’ perceptions of the interventions. Participants were asked which elements of the intervention continued to influence personal and work life after the active intervention period, and which delivery mode was perceived as effective for well-being support. Along with the quantitative data, these open-ended responses were used to guide the development of the semi-structured interview guide for qualitative data collection.

#### Data analysis

Quantitative data were analyzed using a linear mixed model to assess the effect of each intervention arm on each outcome across time points. Batch, gender, practice setting, and years of experience were included as covariates to account for any pre-existing differences in outcome measures. Because of the small sample size of participants practicing in university hospitals or academic medical centers (*n* = 2), they were excluded from the analysis due to their under-representation and distinct job description (research/teaching duties), to ensure a more comparable sample of clinical practitioners. Additional planned contrasts (∆*EMM*_*group*_ = *EMM*_*groupT2*_ – *EMM*_*group T1*_) were conducted to identify the simple time effect of each group. Internal consistency of multi‑item scales was assessed using Cronbach’s α and McDonald’s ω at each time point; values ≥ 0.70 were considered acceptable.

### Qualitative component

#### Data collection

To explore in-depth the experience and challenges of participants of the intervention groups, they were invited to volunteer for semi-structured interviews. Among the volunteers, purposive sampling was carried out to allow for data across all variables including, gender, trainee/fellow, public/private practice, and experimental group (Exp1/Exp2). The interview guide (*in Supplementary Information*) was developed by AC and WKC based on the research question, relevant literature and quantitative results, containing open-ended questions to explore the desired concepts. The research team further reviewed the interview guide to include probe questions to clarify ambiguities and to better explore perspectives.

The interview was conducted through videotelephony software Zoom™ (Zoom Video Communication Inc., San Jose, California) by AC, each lasted approximately 30 min. Only the audio of the interviews was recorded and stored in a cloud server under password protection. The interviews were conducted in both English and Cantonese depending on the preference of the interviewee, to maintain authenticity and ensure ease of conversation. The audio recordings were pseudonymized and transcribed verbatim for subsequent analysis. Throughout the research process, the principal investigator (AC) performed peer debriefing meetings with CC to clarify and consolidate understanding of themes that emerged from the interviews. AC and CC also engaged in reflexivity discussions to ensure transparency and ethical conduct throughout the research process. The interview transcripts were analysed concurrently with data collection between AC and CC to identify areas where the interviews could be improved for deeper and more meaningful discussion. Sampling continued until code saturation was reached [59].

#### Data analysis

Framework analysis was conducted according to Gale et al. [60] Data analysis occurred concurrently with data collection, and constant comparisons were made. Transcripts were independently reviewed by AC and CC and analysed. Through a systematic and iterative process of reading the transcripts for familiarization, identification and noting of initial ideas, the researchers generated initial codes after the first three interviews. The two sets of codes were compared and discussed in order to resolve and combine into conceptually similar themes. This iterative process continued throughout the qualitative interviews, and new emerging themes were either documented or incorporated into existing themes and the descriptors revised. The process occurred concurrently with the interviews until code saturation was reached. Next, the themes were reviewed by the researchers and checked against the coded extracts, entire data set, and the theoretical constructs of PERMA theory of wellbeing [21], broaden and build [44], self-compassion [43] and high quality connections [61].

Qualitative data analysis software (ATLAS.ti v8, Scientific Software Development GmbH, Berlin, Germany) was used to code all transcripts against the themes. Interviews conducted in Cantonese were transcribed in the original language and then translated into English for the purpose of this manuscript. All translations were verified by the bilingual research team (AC and CC) to ensure conceptual equivalence and semantic accuracy.

#### Rigor and trustworthiness

Member checking was performed with sampled interviewees to ensure validity of the qualitative results. Peer debriefing was performed throughout to ensure credibility of data based on the planned analysis of the research team. Memos were kept as a method of audit trail when new ideas or themes emerged.

## Results

Overall demographics of participants are shown in Table 2. A total of 101 participants were recruited, with 53 in Batch 1 and 48 in Batch 2. 54% of participants were male and 46% were female, while majority were fellows (68%) and worked in the public sector (70%). Mean exercise completion rates were 78% for Exp1 and 59% for Exp2.

### Quantitative phase

#### Primary outcomes

**Fig. 2:**
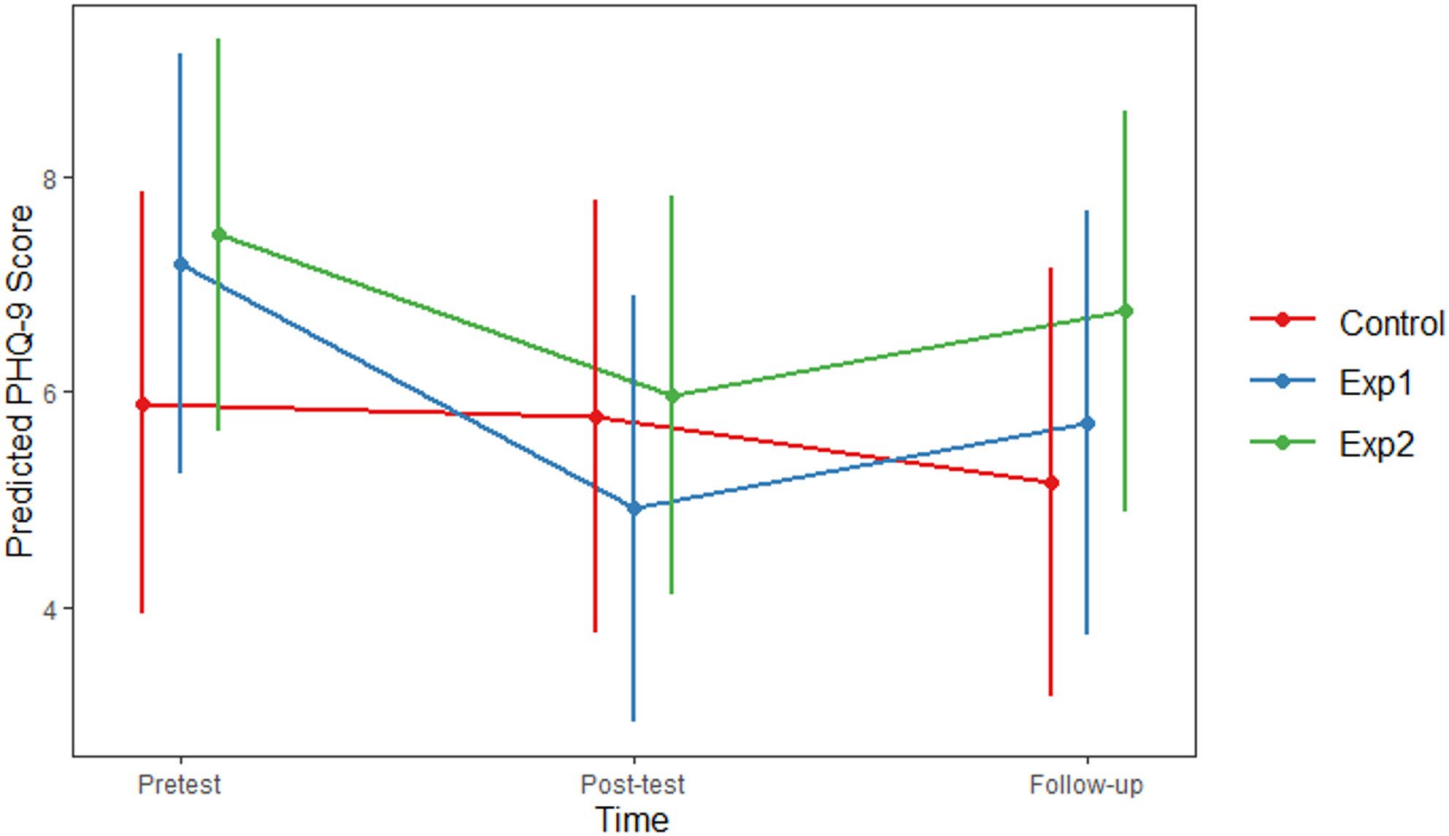
Predicted group mean of PHQ-9 across time points. Note. The mean is averaged across gender and practice setting, and years of experience were set at “5–9 years”

The results of the linear mixed models of each primary outcome are presented in Table 3. Reliability measures of each subscale are provided in Table 4, indicating the internal consistency for all outcomes scales was good to excellent, with Cronbach’s α and McDonald’s ω generally ranging from approximately 0.74 to 0.96 across groups and time points. None of the primary outcomes changed significantly across the three time points (pre, post and 1 year follow up) with or without intervention, except for client-related burnout and perceived stress, both of which increased at post-test. There was no significant post-intervention improvement in the primary outcomes immediately or one year after taking either module, except for depressive symptoms measured by PHQ-9, which showed a significant decrease immediately after completing Exp1, *β* = -2.13, 95% *CI* [-4.19, -0.07], *t* = -2.02, *p* = 0.045. This change was not sustained at the 1-year follow-up (Fig. 2).

**Table 3:** Linear mixed model of primary outcomes

	PERMA Overall	GAD-7	PHQ-9	PSS-10
(Intercept)	6.86 ^***^	5.57 ^***^	3.90 ^***^	20.30 ^***^
	[6.33, 7.39]	[3.81, 7.33]	[1.77, 6.03]	[18.68, 21.91]
Batch	0.10	-0.63	-0.05	-1.21 ^*^
	[-0.32, 0.51]	[-1.94, 0.68]	[-1.69, 1.59]	[-2.36, -0.07]
Gender	-0.25	0.47	0.06	0.40
	[-0.64, 0.15]	[-0.78, 1.73]	[-1.51, 1.63]	[-0.70, 1.49]
Practice Setting	-0.57	1.98 ^*^	2.11	-0.48
	[-1.14, 0.00]	[0.15, 3.82]	[-0.17, 4.39]	[-2.09, 1.14]
Years of Experience	0.41 ^**^	-1.67 ^***^	-1.76 ^***^	-0.40
	[0.18, 0.65]	[-2.41, -0.92]	[-2.69, -0.82]	[-1.06, 0.26]
Post-test	0.22	0.02	-0.13	1.66 *
	[-0.11, 0.54]	[-1.43, 1.48]	[-1.63, 1.37]	[0.09, 3.22]
Follow-up	0.22	-1.28	-0.73	-0.51
	[-0.09, 0.54]	[-2.69, 0.13]	[-2.18, 0.72]	[-2.03, 1.01]
Exp Group 1	-0.09	0.51	1.29	0.80
	[-0.63, 0.44]	[-1.34, 2.36]	[-0.89, 3.48]	[-0.96, 2.56]
Exp Group 2	0.15	0.02	1.50	0.43
	[-0.37, 0.67]	[-1.79, 1.82]	[-0.62, 3.63]	[-1.29, 2.14]
Post-test: Exp1	0.21	-1.07	-2.13 *	-1.62
	[-0.24, 0.66]	[-3.07, 0.93]	[-4.19, -0.07]	[-3.77, 0.53]
Post-test: Exp2	0.08	-0.16	-0.75	-0.54
	[-0.36, 0.52]	[-2.11, 1.79]	[-2.76, 1.27]	[-2.64, 1.56]
Follow-up: Exp1	-0.07	-0.82	-1.36	-2.02
	[-0.51, 0.37]	[-2.78, 1.14]	[-3.38, 0.66]	[-4.12, 0.09]
Follow-up: Exp2	0.00	0.90	-0.02	-0.86
	[-0.43, 0.43]	[-1.02, 2.81]	[-2.00, 1.96]	[-2.92, 1.20]
*N*	208	206	206	206
*N* (ID)	74	74	74	74
AIC	503.13	1029.83	1067.06	1031.15
BIC	553.20	1079.75	1116.98	1081.07
*R*^*2*^ (fixed)	0.14	0.17	0.17	0.11
*R*^*2*^ (total)	0.72	0.55	0.66	0.38
(Intercept)	49.53 ***	49.63 ***	61.13 ***	
	[41.60, 57.47]	[45.51, 53.74]	[52.97, 69.30]	
Batch	4.13	0.92	-0.25	
	[-2.01, 10.27]	[-2.06, 3.89]	[-6.62, 6.11]	
Gender	-4.10	-0.86	-2.60	
	[-9.96, 1.75]	[-3.71, 1.98]	[-8.67, 3.47]	
Practice Setting	-4.69	-1.36	-6.44	
	[-13.17, 3.79]	[-5.53, 2.81]	[-15.22, 2.33]	
Years of Experience	5.34 **	2.66 **	2.50	
	[1.86, 8.82]	[0.96, 4.37]	[-1.10, 6.11]	
Post-test	0.25	2.54	5.53 *	
	[-5.28, 5.78]	[-1.21, 6.30]	[0.15, 10.91]	
Follow-up	3.29	0.78	1.86	
	[-2.05, 8.63]	[-2.86, 4.41]	[-3.33, 7.05]	
Exp Group 1	1.14	0.20	6.21	
	[-7.00, 9.28]	[-4.22, 4.63]	[-2.10, 14.52]	
Exp Group 2	0.39	3.39	-0.56	
	[-7.53, 8.30]	[-0.92, 7.70]	[-8.64, 7.52]	
Post-test:Exp1	5.44	3.06	-3.26	
	[-2.10, 12.98]	[-2.07, 8.19]	[-10.60, 4.07]	
Post-test:Exp2	0.57	0.95	-3.95	
	[-6.84, 7.97]	[-4.10, 5.99]	[-11.15, 3.26]	
Follow-up:Exp1	2.68	-2.27	-2.16	
	[-4.76, 10.11]	[-7.33, 2.78]	[-9.39, 5.07]	
Follow-up:Exp2	0.71	0.17	3.38	
	[-6.52, 7.93]	[-4.75, 5.09]	[-3.65, 10.41]	
*N*	208	208	208	
*N* (ID)	74	74	74	
AIC	1587.14	1393.01	1584.80	
BIC	1637.20	1443.07	1634.87	
*R2* (fixed)	0.15	0.13	0.07	
*R2* (total)	0.65	0.45	0.66	

Exp1 = Experimental group 1; Exp2 = Experimental group 2. All continuous predictors are mean-centered and scaled by 1 standard deviation. The outcome variable is in its original units

^***^p< 0.001;^**^p< 0.01;^*^p< 0.05

**Table 4:** Reliability measures of outcome measures

Outcome Variable	Time	Control			Exp1			Exp2	
		α	ω		α	ω		α	ω
PERMA	Pretest	0.95	0.96		0.91	0.92		0.83	0.87
	Post-test	0.93	0.94		0.91	0.93		0.91	0.92
	Follow-up	0.93	0.94		0.91	0.93		0.90	0.92
GAD-7	Pretest	0.85	0.85		0.86	0.86		0.79	0.80
	Post-test	0.86	0.87		0.88	0.89		0.74	0.73
	Follow-up	0.85	0.87		0.85	0.85		0.85	0.86
PHQ-9	Pretest	0.82	0.83		0.79	0.83		0.88	0.88
	Post-test	0.85	0.86		0.63	0.62		0.9	0.91
	Follow-up	0.84	0.86		0.79	0.81		0.92	0.92
PSS-10	Pretest	0.85	0.86		0.84	0.84		0.82	0.84
	Post-test	0.81	0.83		0.81	0.81		0.81	0.81
	Follow-up	0.88	0.88		0.75	0.77		0.82	0.85
CBI - Personal	Pretest	0.85	0.85		0.88	0.88		0.84	0.84
	Post-test	0.83	0.85		0.83	0.84		0.78	0.79
	Follow-up	0.89	0.89		0.88	0.88		0.75	0.77
CBI - Work-related	Pretest	0.87	0.88		0.79	0.80		0.84	0.85
	Post-test	0.74	0.76		0.80	0.81		0.84	0.84
	Follow-up	0.86	0.87		0.86	0.87		0.81	0.83
CBI - Client-related	Pretest	0.86	0.87		0.78	0.84		0.86	0.87
	Post-test	0.85	0.87		0.81	0.85		0.84	0.86
	Follow-up	0.84	0.88		0.86	0.88		0.81	0.84
SCS	Pretest	0.82	0.82		0.84	0.85		0.81	0.82
	Post-test	0.86	0.86		0.78	0.78		0.75	0.74
	Follow-up	0.86	0.86		0.88	0.88		0.83	0.84
WMI	Pretest	0.92	0.93		0.83	0.83		0.89	0.90
	Post-test	0.78	0.82		0.79	0.83		0.82	0.84
	Follow-up	0.90	0.91		0.88	0.89		0.92	0.93
WGS	Pretest	0.85	0.85		0.83	0.84		0.88	0.88
	Post-test	0.85	0.86		0.89	0.91		0.88	0.90
	Follow-up	0.91	0.91		0.88	0.90		0.88	0.89

#### Secondary outcomes

**Fig. 3:**
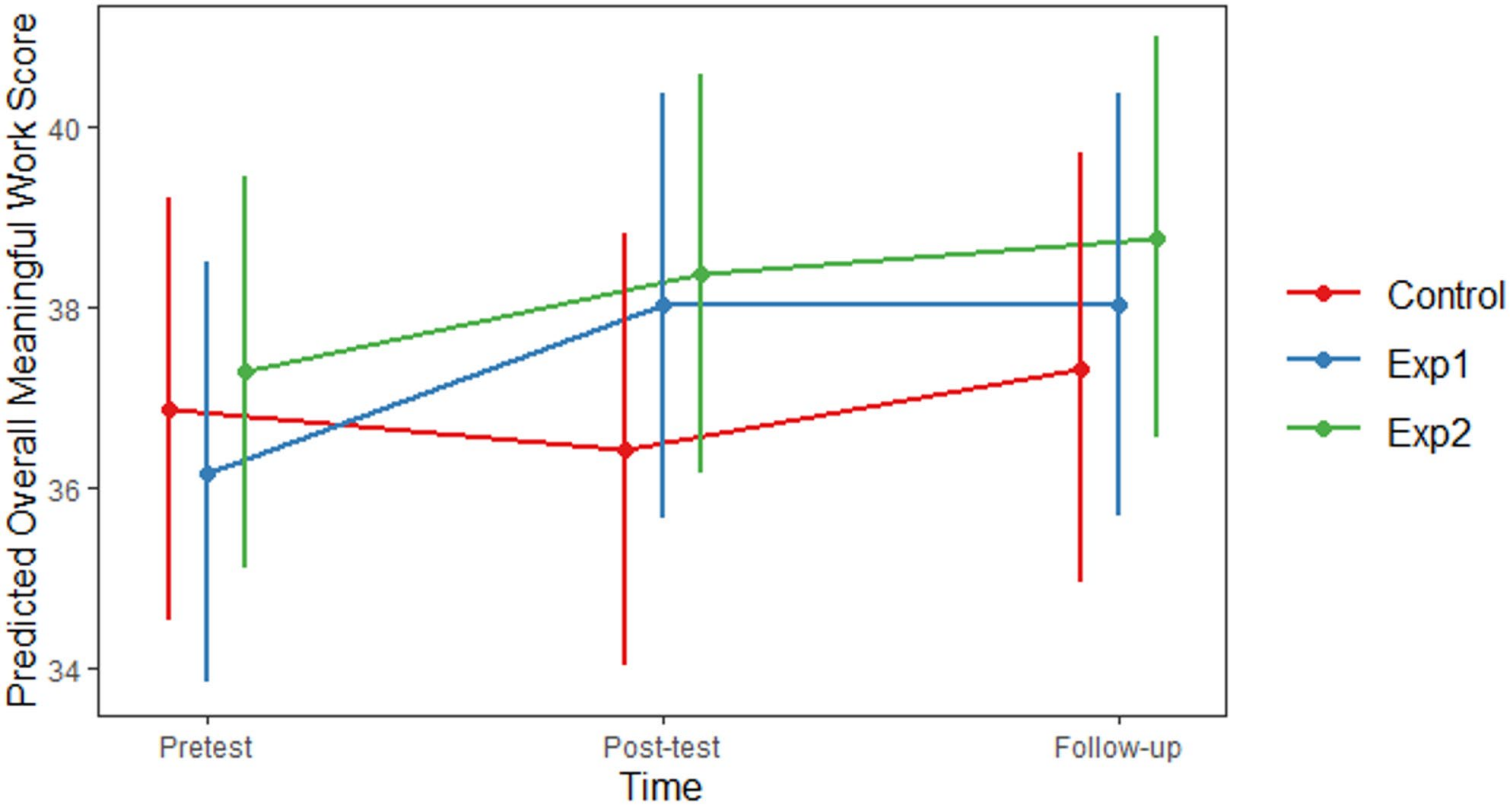
Predicted group mean of WMI across time points. Note. The mean is averaged across gender and practice setting, and years of experience were set at “5–9 years”

The results of the linear mixed models of each secondary outcome are presented in Table 5. Participants in Exp1 showed a significant increase in meaningful work at post-test, *β* = 2.32, 95% *CI* [0.08, 4.55], *t* = 2.03, *p* = 0.044, but the effect was not sustained in the 1-year follow-up (Fig. 3). The other measures did not show significant changes across the three time points, with or without intervention.

**Table 5:** Linear mixed model of secondary outcomes

	SCS	WMI	WGS
(Intercept)	3.38 ^***^	39.69 ^***^	5.46 ^***^
	[3.10, 3.66]	[37.13, 42.24]	[5.09, 5.84]
Batch	-0.04	0.69	-0.02
	[-0.25, 0.18]	[-1.31, 2.69]	[-0.31, 0.27]
Gender	-0.26 ^*^	-0.66	-0.03
	[-0.47, -0.05]	[-2.57, 1.25]	[-0.31, 0.24]
Practice Setting	-0.44 ^**^	-3.47 ^*^	-0.44 ^*^
	[-0.74, -0.14]	[-6.23, -0.72]	[-0.84, -0.04]
Years of Experience	0.30 ^***^	1.58 ^**^	0.33 ^***^
	[0.18, 0.43]	[0.45, 2.72]	[0.17, 0.49]
Post-test	0.14	-0.46	0.15
	[-0.08, 0.35]	[-2.10, 1.18]	[-0.13, 0.42]
Follow-up	0.19	0.45	0.05
	[-0.02, 0.40]	[-1.13, 2.04]	[-0.22, 0.31]
Exp Group 1	-0.16	-0.69	0.07
	[-0.46, 0.13]	[-3.28, 1.90]	[-0.31, 0.46]
Exp Group 2	-0.10	0.31	0.23
	[-0.39, 0.19]	[-2.21, 2.82]	[-0.14, 0.61]
Post-test: Exp1	-0.07	2.32^*^	-0.02
	[-0.36, 0.23]	[0.08, 4.55]	[-0.40, 0.35]
Post-test: Exp2	-0.04	1.41	0.02
	[-0.33, 0.25]	[-0.79, 3.60]	[-0.35, 0.39]
Follow-up: Exp1	0.04	1.54	0.14
	[-0.25, 0.33]	[-0.66, 3.75]	[-0.23, 0.51]
Follow-up: Exp2	0.06	0.98	0.23
	[-0.22, 0.35]	[-1.18, 3.14]	[-0.13, 0.59]
*N*	206	207	206
*N* (ID)	74	74	74
AIC	305.74	1120.30	404.33
BIC	355.66	1170.29	454.25
*R*^*2*^ (fixed)	0.24	0.11	0.18
*R*^*2*^ (total)	0.65	0.69	0.64

Exp1 = Experimental group 1; Exp2 = Experimental group 2. All continuous predictors are mean-centered and scaled by 1 standard deviation. The outcome variable is in its original units

^***^ *p* < 0.001; ^**^ *p* < 0.01; ^*^ *p* < 0.05

### Qualitative phase

Nine participants consented to engage in interviews (Table 6). 67% of the interviewees were fellows, while 78% worked in public hospitals. No new codes or themes emerged after the 7th interview, and analysis of subsequent interviews confirmed thematic saturation.

**Table 6:** Qualitative interviewee demographics

Interviewee	Gender	Fellow/Trainee	Public/Private	Exp1/Exp2
P1	M	Fellow	Private	Exp1
P2	M	Trainee	Public	Exp2
P3	F	Fellow	Public	Exp2
P4	F	Fellow	Public	Exp1
P5	F	Fellow	Public	Exp1
P6	M	Trainee	Public	Exp1
P7	M	Trainee	Public	Exp2
P8	F	Trainee	Public	Exp2
P9	M	Fellow	Public	Exp2

After analysis of 9 semi-structured interviews, 6 main themes were identified relating to the feasibility of positive psychology interventions via a web-based platform, experiences of the intervention, and the impact of positive psychology on personal and professional lives. The 6 themes included: (1) motivation for adoption of positive psychology interventions (2) web-based intervention design implications (3) relational building through positive psychology (4) contextual implications to sustaining positive psychology practice (5) transcendence between personal and professional domains, and (6) institutional validation for sustainability.

### Theme 1: motivation for adoption of positive psychology interventions

Adherence and sustained practice were fundamentally enabled by participants’ intrinsic desire for psychological improvement and the perceived high utility of the skills, resulting in a deep internal shift toward emotional self-management. Participants were driven by a core aspiration for change and acknowledged that the voluntary nature of the program demanded self-motivation. Those who completed the most modules in the program were self-motivated to learn more about positive psychology and how to apply it in professional and personal lives – “my main motivation comes from the perception that it will help me overcome challenges, and also if I feel that it not only helps me, but also those around me, I am more engaged in the activities.” (P4).

Nevertheless, the greatest motivator was the cultivation of self-regulation through cognitive re-appraisal, consistent with the broaden-and-build theory [44], as well as perceived applicability of positive psychology. This included the conversion of negative self-thought to positive self-talk in stressful clinical situations, and the practice of self-compassion. While one participant noted that, “when faced with challenging cases, it is easy for us to be anxious, and have negative thoughts such as ‘I can’t handle it’, or being anxious about risks and complications. But if I could calm down and have positive self-talk and reaffirm that I have managed similar or even more challenging cases... I can stop myself from having these spiraling negative thoughts.” (P1); another participant realized the importance of self-compassion – “I have low sense of self... but... you have to treat yourself better, if you don’t treat yourself better, how do you treat others well?” (P4).

### Theme 2: web-based intervention design implications

The web-based learning mode with bite-sized exercises interlaced with WhatsApp discussions, was praised for its flexibility, as one participant noted, “I think they were quite bite-sized, so I can finish them like in 5 minutes… so it’s a bit more interactive instead of just reading… it engaged me more.” (P2) Nonetheless, there were some other challenges to engagement. Notably, the participation followed a pattern of initial active engagement and discussion, which gradually waned as clinical duties or personal life events (such as weddings or examinations) took priority. As one participant noted, “if it’s very busy… it might just go to the back of my head and I forget about it” (P2). Moreover, a few participants commented that the links to web-based exercises were often lost in the sea of WhatsApp discussions, making it challenging to access and review previously posted material.

As for the WhatsApp discussions and sharing, lack of psychological safety and concern for hierarchy were challenges for some participants, as there were a mixture of people that they may or may not be familiar with, and particularly “afraid to share with senior people in the group” (P9). Despite this, participants felt that the shared vulnerability of those who were willing to share their experiences allowed participants to appreciate that “we are more similar than we thought. despite with different seniority and background” (P8), and vicariously learn – “learning from having to share your own thoughts and then listening to other people’s thoughts was maybe what I remember most about the whole experience.” (P3).

### Theme 3: relational building through positive psychology

Positive psychology was successfully transferred into relational practice, moving beyond internal change to shape the workplace culture through affirmation, gratitude, and empathy, thus building high-quality connections. The practice of fostering a positive environment through expressing gratitude to colleagues such as “show appreciation to my colleagues, including nurses” (P8), and giving positive feedback “a little bit of recognition, validation” (P6) has created psychological safety and “encourages them a little bit to open up to me personally when they come into work, when they ask questions, and show their feelings” (P6). It has also enabled a culture of mutual support in the workplace, as “we learned to encourage each other” (P5).

### Theme 4: contextual implications to sustaining positive psychology practice

While participants appreciated that the interventions were focused on anaesthesiologists and “brought a lot of relevance because we spend a lot of time in the workplace” (P2), there was consensus that in the heat of critical clinical moments, application of positive psychology techniques was challenging. As one participant noted, there is “not enough time, or I feel pressured. like for example in a critical situation, I simply do not have the headspace to process. That’s probably quite a significant obstacle.” (P7) Most often, “after a few days, after things have settled, I am more calm. then would apply these techniques.” (P5).

For sustained change and application to the workplace, conscious efforts and practice are instrumental. “Actually when I first learned these techniques, I really needed to consciously remind myself… but with time, the mindset has changed...” “for example gratitude with 3 good things... I don’t have to consciously make an effort to do it anymore... gratitude really can be learned.” (P5).

### Theme 5: transcendence between personal and professional domains

The positive psychology intervention skills demonstrated powerful transcendence and transfer, showing positive effects both in the workplace (previous themes) and in personal life, particularly with family relationships. “In the past, I am frustrated at my < family member>, as < they > did not meet expectations. However, I reflected and realized that even if < they> moved forward one small step, it was already a large improvement… Instead of reprimanding < them>, I showed more appreciation and encouraged < them>… it really helped because < they were> happier, and < they> felt that I could be trusted.” (P4) One participant was even able to share these techniques with a depressed family member and observed a positive change in emotions (P5), demonstrating a “ripple” effect of these positive psychology interventions.

### Theme 6: institutional validation for sustainability

Participants appreciated the novelty of promulgation of positive psychology interventions through the Hong Kong College of Anaesthesiologists – “someone cares about our psychological wellbeing” (P8), and this institutional initiative addresses the crucial, unspoken need, as another participant stated that “no one has taught us how to be positive in the workplace… and if done well, the whole culture will be more positive, everyone will be happier and more meaningful…”(P9) However, it calls into question the sustainability, as there is perceived to be “no stake” (P6) in implementing such practices in the workplace. Some participants suggested promoting the program as essential professional training (P9), and even incorporating them early in anaesthesia training (P5) such that there is early exposure to positive psychology skills.

## Discussion

This mixed-methods explanatory sequential feasibility randomized controlled trial showed that delivery of web-based positive psychology interventions to anaesthesiologists was feasible and acceptable, although it was not powered to provide definitive evidence of efficacy. Feasibility was demonstrated by the overall completion rate exceeding 50% in both intervention groups, and acceptability was corroborated by the qualitative data. As planned, quantitative analyses of psychological outcomes were conducted as exploratory efficacy assessments: most results did not demonstrate significant between-group differences in the primary outcomes, but there was a signal that the individual-oriented intervention (Exp1) was associated with a within-group decrease in depression immediately post-intervention.

In the overall sample, we observed a small increase in client-related burnout and perceived stress post-intervention. Given the study’s feasibility design, these findings should be interpreted cautiously. Possible explanations include heightened awareness as participants engaged in reflective PPIs, and concurrent contextual stressors (e.g. service pressures, examinations, rota changes) that were not controlled for. For secondary outcomes, there was significant increase in meaningful work for the individual-oriented intervention group immediately (Exp1) post-intervention, although this effect was not sustained at 1-year follow up.

Self-determination theory (SDT), as developed by Ryan and Deci [62], provides a relevant theoretical lens for understanding why positive psychology interventions (PPIs) may influence doctors’ well-being in the clinical environment [63], and for elucidating potential mechanisms underlying the observed differences between individual-oriented and workplace-oriented PPIs [31]. SDT posits that three inherent psychological needs – autonomy, competence, and relatedness – are vital to motivation and well-being. This framework aligns conceptually with PERMA-based positive psychology, in that positive emotions and engagement may be supported through autonomy; relationships reflect relatedness; meaning and accomplishment may reinforce competence [63, 64]. The interface between SDT and positive psychology has been well-described [63, 64], and provides a conceptual rationale for using SDT to interpret our PPI findings.

Environments that support self-determination, especially autonomy, lead to positive health and wellness outcomes [65, 66]. In medicine, clinical environments tend to be hierarchical and psychologically unsafe, and leaders and clinician educators often have not received adequate training on how to support learners’ basic psychological needs [67]. Against this theoretical backdrop, our findings help to explain the distinct patterns observed across the two intervention arms. Contrary to a previous systematic review [31], in our cohort, individual-oriented PPI in Exp1, which focused on self-awareness, compassion and leadership, appeared to lead to short-term improvements in depression and meaningful work. One possible explanation is that such PPIs may have supported autonomy and competence in the face of clinical situations (Theme 1), as well as relatedness through fostering positive relationships (Theme 3), thereby helping to fulfil core psychological needs and support well-being. In the context of anaesthesiology practice in Hong Kong, clinicians often work in relative professional isolation and are exposed to sustained high-stakes demands, including the care of critically ill patients, stringent performance standards, perfectionistic norms, and low tolerance for error. Within such a need-thwarting environment, characterised by constrained autonomy and threats to perceived competence and relatedness, PPIs that build psychological resources, such as positive emotion, cognitive reappraisal and self-compassion, may be particularly important for supporting individuals despite adversity.

By contrast, workplace-oriented PPI, which targeted rapport building, conflict management, work accomplishment and meaning, may have had less of an effect in our cohort due to the nature of anaesthesia practice in our locality. The difference in mean module completion rates between Exp1 (78%) and Exp2 (59%) also seems to reflect the acceptability of individual-oriented compared with workplace-oriented PPIs. In Hong Kong, particularly in the public sector, anaesthesiologists frequently occupy a behind-the-scenes role; work demands are largely structured around surgical priorities and service demands; and there is often limited recognition from patients and healthcare teams. As such, these PPIs may have been experienced as extraneous tasks rather than autonomy-support resources since the aforementioned challenges are shaped by the broader working environment and culture, making the content difficult to internalize and apply during busy clinical duties (Theme 4).

Nevertheless, despite the seemingly positive effects of individual-oriented PPI in our cohort, the effects were not sustained. Anaesthesiologists in Hong Kong, particularly in the public sector, are faced with the challenges of rigorous specialty exams, chronic understaffing, hierarchical work cultures and unpredictable work hours – all of which threaten the autonomy of anaesthesiologists and possibly dilute the effect of the PPI in the long term. Yet, the triangulation of results may point to several considerations when evaluating or implementing such interventions in the future.

Firstly, when individuals were able to internalize the tools of positive psychology, in keeping with SDT [65], they were intrinsically motivated to translate them into practice when faced with personal and workplace challenges (Theme 1 and 4). To facilitate such internalization, future PPIs should be explicitly designed to highlight their relevance to clinical practice and clearly link exercises to common stressors and role demands, as well as encourage participants to adapt practices and apply them to their own circumstances. This may help overcome the commonly identified implementation barriers such as heavy workload or life events [30]. Secondly, the PPIs should be directed at a system level, such that autonomy-support environments (Theme 6) are created to empower individuals to flourish in a sustainable manner [31, 68–70]. These may include increasing clinician input into workflows, training leaders in autonomy-supportive behaviours, staff recognition for contributions to patient care, and embedding meaning-focused practices into daily routines, rather than solely relying on individual-level interventions. Thirdly, bite-sized web-based PPIs are feasible and practicable (Theme 2), but should be redesigned using SDT-informed strategies, such as offering participants greater choices over which modules they engage in, framing activities to make them more relevant to the individual growth and applicable to workplace challenges (Theme 4), and providing psychologically safe environments for them to engage in such activities (Theme 2).

### Implications and recommendations

The findings from this study have implications for postgraduate medical education, particularly workplace learning and continuous professional development (CPD) in anaesthesia. Well-being initiatives, such as PPIs, should be integrated into training curricula to help residents build psychological resources to manage burnout and maintain well-being throughout their careers (Theme 6). This has shown some promise in anaesthesia residency programs that have implemented wellness curricula [71, 72]. To advance this work, SDT principles should be explicitly applied in the design of PPIs such that they are contextualized to workplace demands, actively support autonomy, and are integrated with personal growth and professional identity development in anaesthesia practice.

Of note, evidence for implementation of PPIs within CPD for practicing clinicians are limited [31]. However, our cohort provides preliminary, exploratory evidence that PPIs may have a positive effect on the well-being for anaesthesiologists in Hong Kong. To facilitate integration into busy clinical schedules, web-based bite-sized interventions may be feasible, provided that they are clearly relevant to clinicians’ challenges, and offer autonomy in the selection and adaptation of modules. Finally, to make the effects of well-being initiatives sustainable, individual-level PPIs should be complemented by faculty development [67], system-directed PPIs [31] and system-level organizational changes [68, 73] to create autonomy-supportive, psychologically safe learning and working environments where anaesthesiologists can flourish [69, 70].

### Strengths and limitations

In contrast with systematic reviews that demonstrated potential benefits of positive psychology interventions in healthcare workers, many of the studies evaluated used nonrandomized or quasi-experimental designs, as well as variable outcome assessments [29]. Moreover, long-term follow up data were often lacking, with the longest follow-up period at 3 months. The strength of this study is that not only a randomized-controlled methodology was employed using validated outcome measurement tools, longitudinal assessment across 3 time points (pre, post, and 1 year follow up) was conducted. Moreover, the mixed methods methodology enabled rigorous in-depth qualitative analysis that provided valuable insights into the impact of web-based PPIs on both professional and personal domains of anaesthesiologists [42].

Interventions were grounded in established positive psychology theories [21, 43–46] and culturally-adapted to the context of anaesthesia practice in Hong Kong; while easily accessible web-based technology served as the backbone for participant engagement. Methodological rigor was applied in both quantitative and qualitative components to ensure validity and reliability of the results. Unlike many PPI studies conducted among healthcare workers with heterogeneous professional backgrounds leading to the possibility of confounders [29], our study focused on the feasibility of PPIs in a specific homogeneous group of professionals in Hong Kong.

Some limitations need to be considered. Recruitment proved challenging in this cohort despite institutional support and multiple recruitment strategies, reflecting the realities of anaesthesia practice in Hong Kong. As a result, our sample size (*N* = 102) fell short of the number required for conventional power to detect medium effects, and sensitivity analyses showed that the study was only adequately powered to detect large effects. Also, the results reported multiple outcomes without adjustment for multiple comparisons due to the exploratory nature of our study. Accordingly, this mixed‑methods feasibility randomised controlled trial should be interpreted as an exploratory study that provides implementation insights and preliminary estimates of potential intervention effects to inform the design and sample size of future, larger‑scale trials.

The study relied mainly on self-report measures, and qualitative data from a volunteer subset may overrepresent highly engaged participants. Moreover, similar to the well-documented challenges with randomized controlled trials (RCT) in psychological interventions [74], there were potential significant confounders to well-being, including the dynamic nature of healthcare environments, organizations and workload, as well as life events (marriage, examinations, adverse clinical events, etc.).

Practical implementation issues, including variable psychological safety in group chats, module length, and engagement that largely occurred outside clinical hours, as well as the nature of anaesthesia practice itself, may limit generalisability to other clinical settings and indicate the need for pragmatic adaptations before broader implementation. Larger trials with repeated assessments, observer‑rated outcomes, and embedded process measures to track adherence and practice frequency can identify which specific combinations of interventions could produce sustained effects.

## Conclusion

This explanatory sequential mixed-methods feasibility randomized-controlled study provides preliminary evidence that a web-based positive psychology intervention may be acceptable and practically implementable among anaesthesiologists in Hong Kong. Exploratory quantitative findings suggested encouraging short-term reductions in depressive symptoms and increased work meaning, while qualitative data illuminated intrinsic motivations and contextual barriers affecting engagement. Triangulation of results is consistent with the interpretation that PPIs can support the basic psychological needs of SDT, but may require system-level autonomy support for sustained benefit. The findings suggest that future larger-scale efficacy trials are warranted to further evaluate and quantify the effect of SDT-based designs of PPIs, and to evaluate the role of system-directed interventions and organizational leadership support in facilitating the sustainability of well-being initiatives for healthcare professionals in time-constrained, high pressure environments. 

## Supplementary Information

Supplementary Material 1.

Supplementary Material 2.

## Data Availability

The datasets used and/or analyzed during the current study are available from the corresponding author upon reasonable request.

## Abbreviations

PP: Positive Psychology
PPI: Positive Psychology Intervention
SDT: Self Determination Theory
CPD: Continuous Professional Development
P1, P2, P3…: Participant 1, Participant 2, Participant 3…
HKCA: Hong Kong College of Anaesthesiologists
Exp1: Experimental Group 1
Exp2: Experimental Group 2
RCT: Randomized Controlled Trials
GAD-7: Generalized Anxiety Disorder 7-item
PHQ-9: Patient Health Questionnaire
PSS-10: Perceived Stress Scale
CBI: Copenhagen Burnout Inventory
SCS-SF: Self-Compassion Scale Short Form
WAMI: Work and Meaning Inventory
WGS: Work Gratitude Scale
