# Supplementary material for: Exploring the feasibility of a web-based positive psychology program among anaesthesiologists: an explanatory sequential mixed-methods pilot study
Source: BMC Med Educ. 2026 May 18;26:1120. doi: 10.1186/s12909-026-09459-2 (PMC13352935; doi:10.1186/s12909-026-09459-2)

Supplementary Table 1: *Estimated Marginal Means of Outcome Variables*

| Outcome Variable | Time | Control |  | Exp1 |  | Exp2 |
| --- | --- | --- | --- | --- | --- | --- |
|  |  | *EMM* [95% *CI*] |  | *EMM* [95% *CI*] |  | *EMM* [95% *CI*] |
| PERMA | Pretest | 6.46 [5.96, 6.96] |  | 6.30 [5.80, 6.80] |  | 6.15 [5.66, 6.65] |
|  | Post-test | 6.78 [6.28, 7.28] |  | 6.41 [5.91, 6.91] |  | 6.41 [5.91, 6.90] |
|  | Follow-up | 6.63 [6.13, 7.13] |  | 6.33 [5.83, 6.83] |  | 6.63 [6.17, 7.10] |
|  |  |  |  |  |  |  |
| GAD-7 | Pretest | 7.39 [6.86, 7.91] |  | 7.38 [6.86, 7.91] |  | 7.32 [6.79, 7.84] |
|  | Post-test | 7.50 [6.97, 8.02] |  | 7.74 [7.21, 8.27] |  | 7.92 [7.42, 8.42] |
|  | Follow-up | 7.42 [6.89, 7.95] |  | 7.43 [6.90, 7.95] |  | 7.71 [7.18, 8.23] |
|  |  |  |  |  |  |  |
| PHQ-9 | Pretest | 5.39 [4.54, 6.24] |  | 5.69 [4.84, 6.54] |  | 5.14 [4.30, 5.98] |
|  | Post-test | 5.16 [4.31, 6.00] |  | 4.87 [4.02, 5.71] |  | 4.92 [4.07, 5.77] |
|  | Follow-up | 5.25 [4.40, 6.09] |  | 5.09 [4.24, 5.93] |  | 4.77 [3.92, 5.61] |
|  |  |  |  |  |  |  |
| PSS-10 | Pretest | 20.0 [17.9, 22.1] |  | 19.4 [17.3, 21.5] |  | 19.5 [17.4, 21.6] |
|  | Post-test | 18.2 [16.1, 20.3] |  | 18.0 [15.9, 20.1] |  | 20.6 [18.5, 22.6] |
|  | Follow-up | 18.2 [16.1, 20.3] |  | 20.0 [17.8, 22.1] |  | 18.4 [16.3, 20.5] |
|  |  |  |  |  |  |  |
| CBI - Personal | Pretest | 45.4 [39.1, 51.7] |  | 48.0 [41.7, 54.3] |  | 46.4 [40.1, 52.7] |
|  | Post-test | 44.5 [38.2, 50.8] |  | 43.1 [36.8, 49.4] |  | 42.6 [36.3, 48.9] |
|  | Follow-up | 48.3 [42.0, 54.6] |  | 43.3 [37.0, 49.6] |  | 48.1 [41.8, 54.4] |
|  |  |  |  |  |  |  |
| CBI - Work-related | Pretest | 47.7 [43.0, 52.4] |  | 47.3 [42.6, 52.0] |  | 47.9 [43.2, 52.6] |
|  | Post-test | 46.9 [42.2, 51.6] |  | 47.5 [42.8, 52.2] |  | 49.3 [44.6, 54.0] |
|  | Follow-up | 51.5 [46.8, 56.2] |  | 47.4 [42.7, 52.1] |  | 51.7 [47.0, 56.4] |
|  |  |  |  |  |  |  |
| CBI - Client-related | Pretest | 58.6 [53.0, 64.2] |  | 58.8 [53.2, 64.4] |  | 56.6 [51.0, 62.2] |
|  | Post-test | 58.7 [53.1, 64.3] |  | 60.7 [55.1, 66.3] |  | 56.9 [51.3, 62.5] |
|  | Follow-up | 54.3 [48.7, 59.9] |  | 58.4 [52.8, 64.0] |  | 59.7 [54.1, 65.3] |
|  |  |  |  |  |  |  |
| SCS | Pretest | 3.01 [2.75, 3.28] |  | 3.01 [2.74, 3.28] |  | 2.97 [2.70, 3.24] |
|  | Post-test | 2.87 [2.60, 3.14] |  | 2.79 [2.52, 3.06] |  | 2.91 [2.64, 3.18] |
|  | Follow-up | 2.75 [2.48, 3.02] |  | 2.80 [2.53, 3.07] |  | 2.99 [2.72, 3.25] |
|  |  |  |  |  |  |  |
| WMI | Pretest | 37.1 [35.0, 39.2] |  | 36.9 [34.8, 39.0] |  | 37.3 [35.2, 39.4] |
|  | Post-test | 37.3 [35.2, 39.4] |  | 38.0 [35.9, 40.1] |  | 38.1 [36.0, 40.2] |
|  | Follow-up | 36.8 [34.7, 38.9] |  | 37.3 [35.2, 39.4] |  | 38.6 [36.5, 40.7] |
|  |  |  |  |  |  |  |
| WGS | Pretest | 5.01 [4.67, 5.35] |  | 5.06 [4.72, 5.41] |  | 5.06 [4.72, 5.41] |
|  | Post-test | 5.02 [4.68, 5.36] |  | 5.12 [4.78, 5.46] |  | 5.25 [4.91, 5.59] |
|  | Follow-up | 5.32 [4.98, 5.67] |  | 5.54 [5.20, 5.88] |  | 5.36 [5.02, 5.70] |

Supplementary Table 2: *Estimated Time Changes within Groups and Difference-in-Differences Relative to Control*

| **Measures** | **Contrast** | **Control** | |  | **Exp1** | | | |  | **Exp2** | | | |
| --- | --- | --- | --- | --- | --- | --- | --- | --- | --- | --- | --- | --- | --- |
|  |  | ∆*EMM*_group_ | *p* |  | ∆*EMM*_group_ | *p* | **∆***EMM*_group_**-** ∆EMM_control_ | *p* |  | ∆*EMM*_group_ | *p* | **∆***EMM*_group_**-** ∆EMM_control_ | *p* |
| PERMA | Post-test vs. Pretest | 0.21 | 1.00 |  | **0.43** | **0.04** | 0.21 | 1.00 |  | 0.15 | 1.00 | -0.07 | 1.00 |
|  | Follow-up vs. Pretest | 0.22 | 1.00 |  | 0.30 | 0.34 | 0.08 | 1.00 |  | 0.23 | 0.75 | 0.01 | 1.00 |
| GAD-7 | Post-test vs. Pretest | 0.02 | 1.00 |  | -1.05 | 0.82 | -1.07 | 1.00 |  | -0.80 | 1.00 | -0.82 | 1.00 |
|  | Follow-up vs. Pretest | -1.28 | 0.46 |  | -1.44 | 0.23 | -0.16 | 1.00 |  | -0.33 | 1.00 | 0.95 | 1.00 |
| PHQ-9 | Post-test vs. Pretest | -0.13 | 1.00 |  | **-2.26** | **0.01** | -2.13 | 0.18 |  | -1.49 | 0.19 | -1.36 | 0.76 |
|  | Follow-up vs. Pretest | -0.73 | 1.00 |  | -1.48 | 0.24 | -0.75 | 1.00 |  | -0.71 | 1.00 | 0.02 | 1.00 |
| PSS-10 | Post-test vs. Pretest | 1.65 | 0.24 |  | 0.04 | 1.00 | -1.62 | 0.57 |  | -0.37 | 1.00 | -2.02 | 0.25 |
|  | Follow-up vs. Pretest | -0.51 | 1.00 |  | -1.05 | 0.96 | -0.54 | 1.00 |  | -1.47 | 0.26 | -0.97 | 1.00 |
| CBI-Personal | Post-test vs. Pretest | 0.26 | 1.00 |  | 5.69 | 0.19 | 5.43 | 0.64 |  | 2.95 | 1.00 | 2.69 | 1.00 |
|  | Follow-up vs. Pretest | 3.28 | 1.00 |  | 3.85 | 0.86 | 0.57 | 1.00 |  | 4.26 | 0.54 | 0.98 | 1.00 |
| CBI-Work | Post-test vs. Pretest | 2.54 | 1.00 |  | **5.60** | **0.01** | 3.06 | 0.98 |  | 0.27 | 1.00 | -2.27 | 1.00 |
|  | Follow-up vs. Pretest | 0.77 | 1.00 |  | 1.72 | 1.00 | 0.95 | 1.00 |  | 0.99 | 1.00 | 0.22 | 1.00 |
| CBI-Client | Post-test vs. Pretest | 5.53 | 0.28 |  | 2.26 | 1.00 | -3.27 | 1.00 |  | 3.38 | 1.00 | -2.15 | 1.00 |
|  | Follow-up vs. Pretest | 1.85 | 1.00 |  | -2.09 | 1.00 | -3.94 | 1.00 |  | 5.41 | 0.17 | 3.55 | 1.00 |
| SCS | Post-test vs. Pretest | 0.14 | 1.00 |  | 0.07 | 1.00 | -0.07 | 1.00 |  | 0.17 | 0.48 | 0.04 | 1.00 |
|  | Follow-up vs. Pretest | 0.19 | 0.43 |  | 0.15 | 0.84 | -0.04 | 1.00 |  | 0.23 | 0.11 | 0.04 | 1.00 |
| WMI | Post-test vs. Pretest | -0.46 | 1.00 |  | 1.86 | 0.11 | 2.31 | 0.18 |  | 1.08 | 0.91 | 1.54 | 0.69 |
|  | Follow-up vs. Pretest | 0.45 | 1.00 |  | 1.86 | 0.11 | 1.41 | 0.85 |  | 1.49 | 0.30 | 1.03 | 1.00 |
| WGS | Post-test vs. Pretest | 0.15 | 1.00 |  | 0.12 | 1.00 | -0.02 | 1.00 |  | 0.28 | 0.15 | 0.14 | 1.00 |
|  | Follow-up vs. Pretest | 0.05 | 1.00 |  | 0.07 | 1.00 | 0.02 | 1.00 |  | 0.29 | 0.14 | 0.24 | 0.79 |
| Note. For each measure and each group of contrasts, the *p*-values were adjusted for multiple comparisons using the Bonferroni method (6 contrasts for ∆*EMM*_group_ and four contrasts for ∆*EMM*_group_ - ∆EMM_control_). | | | | | | | | | | | | | |

Supplementary Figure 1: *Predicted group mean of outcome measures across time points*
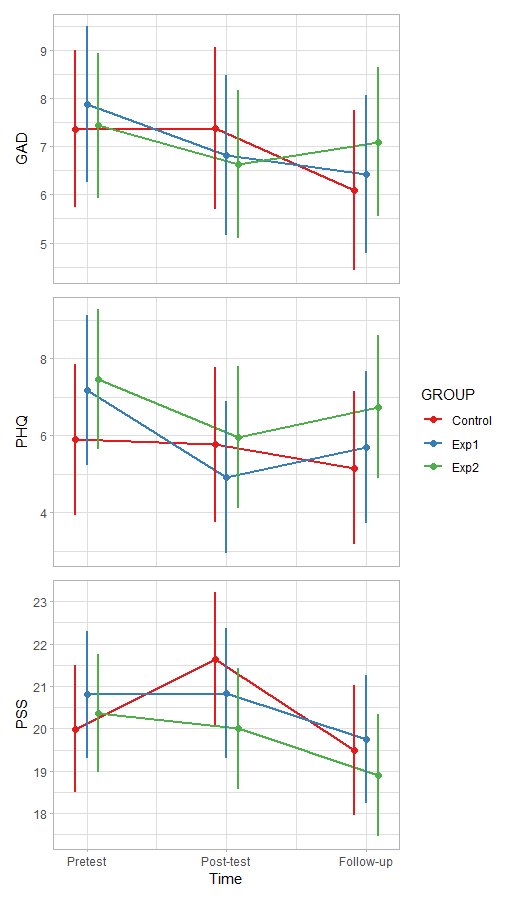


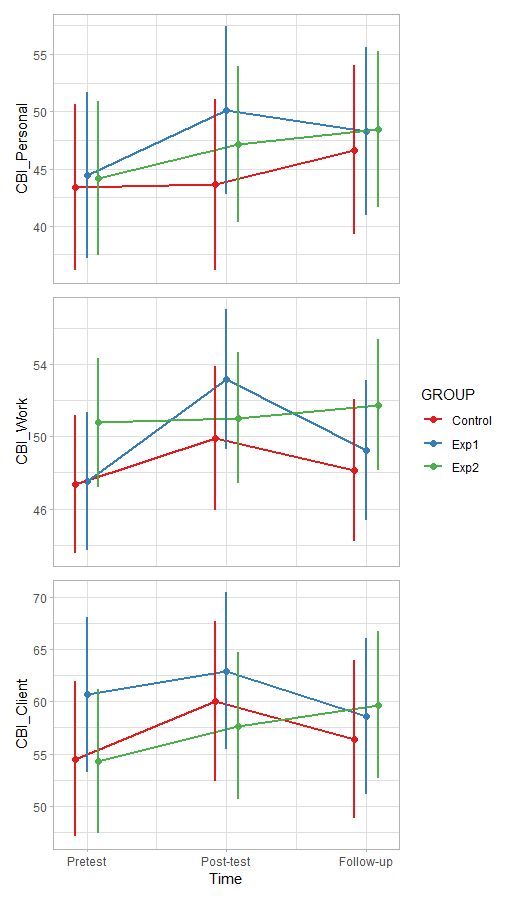


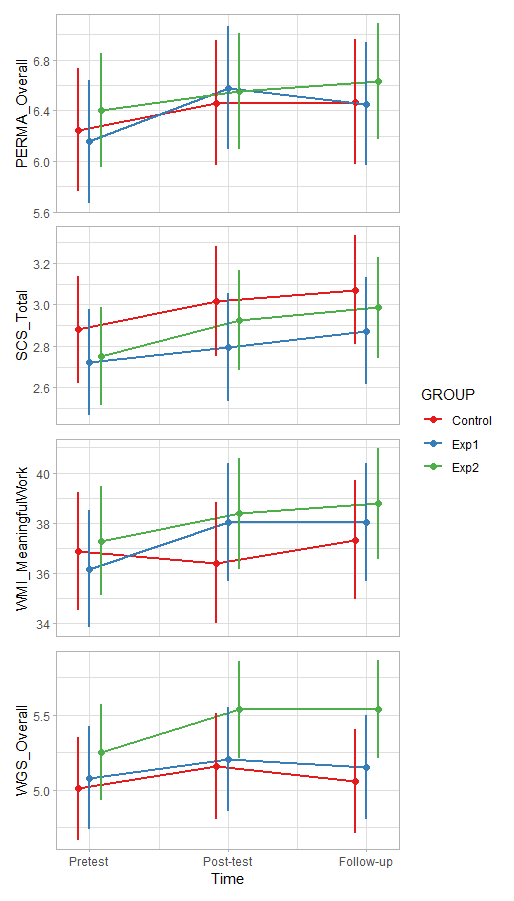

Supplement: Supplementary file 1 — Supplementary Material 1. [file 12909_2026_9459_MOESM1_ESM.docx]
