# Supplementary material for: Exploring the feasibility of a web-based positive psychology program among anaesthesiologists: an explanatory sequential mixed-methods pilot study
Source: BMC Med Educ. 2026 May 18;26:1120. doi: 10.1186/s12909-026-09459-2 (PMC13352935; doi:10.1186/s12909-026-09459-2)
Supplement: Supplementary file 2 — Supplementary Material 2. [file 12909_2026_9459_MOESM2_ESM.docx]

**Supplementary File: Sem-structured Interview Guide**

**Web-based Positive Psychology Study Semi-Structured Interview Guide**

**Guide for the interviewer:**

Before starting the recording and the interview, please obtain consent from all the participants, and let them know the purpose of the interview and the study. Assure them that the interviews are confidential, and the identities of the participants will not be revealed throughout the research process.

Establish ground rules for interview, including confidentiality and mutual respect. The interview can perform some simple icebreaking introductions to help interviewees familiarize with each other.

**Start recording**

**A. Introduction and Rapport-Building (Approx. 5 minutes)**

The interviewer should begin by re-introducing the study, confirming consent, and building rapport.

1. Which cohort did you belong to? Which modules of intervention were you assigned to? Do you work in public or private? Were you a specialist or trainee when you joined the study?
2. Quick recap of the positive psychology intervention
3. "Thank you for speaking with me today. To begin, could you tell me what your overall experience was with the web-based positive psychology program?"
4. "What were your initial expectations when you first signed up for the program?
   - *Probe:* How did the actual experience compare to those initial expectations?"

**B. Programme Adherence and Usage (Approx. 10 minutes)**

This section aims to understand the participant's pattern of use and level of completion.

1. "Thinking about the program's duration, can you describe how consistently you were able to use the platform?"
   - *Probe:* Were there periods when you engaged more or less? What was happening during those times?
2. "Can you walk me through how you integrated the program into your typical week?"
   - *Probe:* Was there a particular time of day or routine that worked best for you? Why?
3. "Which modules or activities in the program did you complete? Which, if any, did you not complete?"
   - *Probe:* What was it about those specific parts that you found most or least engaging?

**C. Challenges Affecting Engagement (Approx. 10-15 minutes)**

This section explores the specific barriers that impacted participation.

1. "What were the primary challenges you faced when trying to engage with the program on a regular basis?"
2. "Let's break that down. Were there any aspects of the program's design or content – such as its usability, the length of modules, or the nature of the exercises – that made it difficult to stay engaged?" (e.g. whatsapp, or online exercises)
3. "How did the demands of your work schedule and environment as an anaesthesiologist affect your ability to participate?"
   - *Probe:* Can you describe how factors like on-call duties, long hours, or workplace stress impacted your engagement?
4. "Were there any personal factors outside of work, such as energy levels or family commitments, that influenced your participation?"
5. "What might have helped you overcome these challenges and engage more fully with the program?"

**D. Applicability and Obstacles in Clinical Practice (Approx. 10-15 minutes)**

This section assesses the real-world application of the learned techniques.

1. "Shifting from the program to the techniques themselves, how applicable did you find the positive psychology skills in your day-to-day professional life?"
2. "Could you share a specific example of a time you attempted to apply a technique you learned at work and personal life?
   - *Probe:* What was the situation, which technique did you use, and what was the outcome?
3. "What are the most significant obstacles you encounter when trying to use these techniques in a clinical setting?"
   - *Probe:* Consider factors like time pressure, workplace culture, team dynamics, or patient interactions.
4. "*From your perspective, how does the applicability of these techniques differ between public and private practice settings?*
   - *Probe: What specific aspects of these different environments make the techniques easier or harder to apply?"*

**E. Closing and Final Thoughts (Approx. 5 minutes)**

This provides an opportunity for final reflections and suggestions.

1. "If you could give one piece of advice to the developers for improving this program for other anaesthesiologists, what would it be?"
2. "Is there anything else about your experience with the program or the techniques that you feel is important for us to know?"
